# The value of preoperative magnetic resonance imaging in predicting postoperative recovery in patients with cervical spondylosis myelopathy: a meta-analysis

**DOI:** 10.6061/clinics/2016(03)10

**Published:** 2016-03

**Authors:** Hui Chen, Jun Pan, Majid Nisar, Huan Bei Zeng, Li Fang Dai, Chao Lou, Si Pin Zhu, Bing Dai, Guang Heng Xiang

**Affiliations:** Second Affiliated Hospital of Wenzhou Medical University, Department of Orthopaedic Surgery, Wenzhou, China

**Keywords:** Cervical Spondylosis Myelopathy, Magnetic Resonance Imaging, Signal Intensity Changes, Meta-Analysis

## Abstract

This meta-analysis was designed to elucidate whether preoperative signal intensity changes could predict the surgical outcomes of patients with cervical spondylosis myelopathy on the basis of T1-weighted and T2-weighted magnetic resonance imaging images. We searched the Medline database and the Cochrane Central Register of Controlled Trials for this purpose and 10 studies meeting our inclusion criteria were identified. In total, 650 cervical spondylosis myelopathy patients with (+) or without (-) intramedullary signal changes on their T2-weighted images were examined. Weighted mean differences and 95% confidence intervals were used to summarize the data. Patients with focal and faint border changes in the intramedullary signal on T2 magnetic resonance imaging had similar Japanese Orthopaedic Association recovery ratios as those with no signal changes on the magnetic resonance imaging images of the spinal cord did. The surgical outcomes were poorer in the patients with both T2 intramedullary signal changes, especially when the signal changes were multisegmental and had a well-defined border and T1 intramedullary signal changes compared with those without intramedullary signal changes. Preoperative magnetic resonance imaging including T1 and T2 imaging can thus be used to predict postoperative recovery in cervical spondylosis myelopathy patients.

## INTRODUCTION

Cervical spondylosis is a degenerative disease in which compression of the spinal cord leads to cervical spondylosis myelopathy (CSM) and neurological deficits. The most common clinical symptoms are gait disturbance, upper limb paresthesias or sensory disturbance and clumsy hands. Intramedullary signal changes (ISCs) in the spinal cord on magnetic resonance imaging (MRI) in cervical compression myelopathy are deemed to reflect pathologic changes in the spinal cord and are regarded as an indicator of the prognosis 1,2,3,4,5,6. Nonetheless, as the cause and the pathogenesis of signal intensity changes within the spinal cord remain unclear, the significance of the signal intensity changes for prognosis remains controversial 7,8,9,10,15,. We thus conducted the present meta-analysis to compare postoperative functional outcomes in CSM patients according to T2-weighted and T1-weighted MRI images.

## MATERIALS AND METHODS

### Search strategy and inclusion criteria

A literature search was performed up to June 2013 using the Medline database and the Cochrane Central Register of Controlled Trials. We screened all fields by combining the terms “cervical spondylotic myelopathy”, “cervical spondylotic”, “cervical compression myelopathy” or “CSM” and “magnetic resonance imaging” or “MRI”. The articles selected were limited to those published in English. A comprehensive search of the reference lists of both the retrieved articles and previous published reviews was also conducted to ensure inclusion of all possible articles.

The following inclusion criteria were used: 1. the study population consisted of patients with CSM who underwent surgery via an anterior, posterior,or combined approach; 2. MRI, including T2-weighted and T1-weighted images, was performed before surgery for all patients; 3. the clinical grade of the CSM was evaluated according to the Japanese Orthopaedic Association (JOA) score for cervical myelopathy before surgery and at the final follow-up, and the postoperative functional assessment was based on the JOA recovery ratio (RR); 4. the study had a comparative design; and 5. the follow-up duration was >6 months. All potential articles were separately reviewed by two authors (X.G.H. and C.H.). Any divergences between the investigators were resolved by discussion.

### Data extraction

Data were collected according to the following categories, where available: 1. basic characteristics, including study design, publication year, inclusion/exclusion criteria, patient age, enrolled number, disease course, surgical modalities and follow-up duration; 2. imaging assessment, consisting of classification of the spinal cord signal pattern; and 3. clinical symptoms, recovery rate, and pre- and postoperative JOA scores. Inconsistencies were resolved through discussion until a consensus was reached.

### Assessment of risk of bias

The studies included in the current analysis were all non-randomized, and we used the MINORS score to assess the risk of bias 32. The main items for MINORS include prospective collection of data, a follow-up period appropriate to the aim of the study, unbiased assessment of the study endpoint, loss to follow-up of less than 5%, baseline equivalence of the groups and adequate statistical analyses. A maximum score of 24 points can be generated for each included comparative study.

### Meta-analysis

In our study, the postoperative JOA RR was used as an evaluation index. Weighted mean differences (WMDs) and 95% confidence intervals (CIs) were used to summarize the data. The level of significance was set at *p*<0.05. The *χ^2^* test and *I^2^* statistic (considered significant when the *p* value for the *χ^2^* test is <0.10 or *I^2^* >50%) were performed to evaluate heterogeneity. Fixed-effect models were applied when the statistical heterogeneity was significant and a random-effect model was applied when the heterogeneity was not significant. Funnel plots were employed to assess the potential publication bias. The analysis was performed using the statistical software Review Manager Version 5.0 (Cochrane Collaboration, Oxford, UK).

## RESULTS

### Literature search

**Figure 1-:**
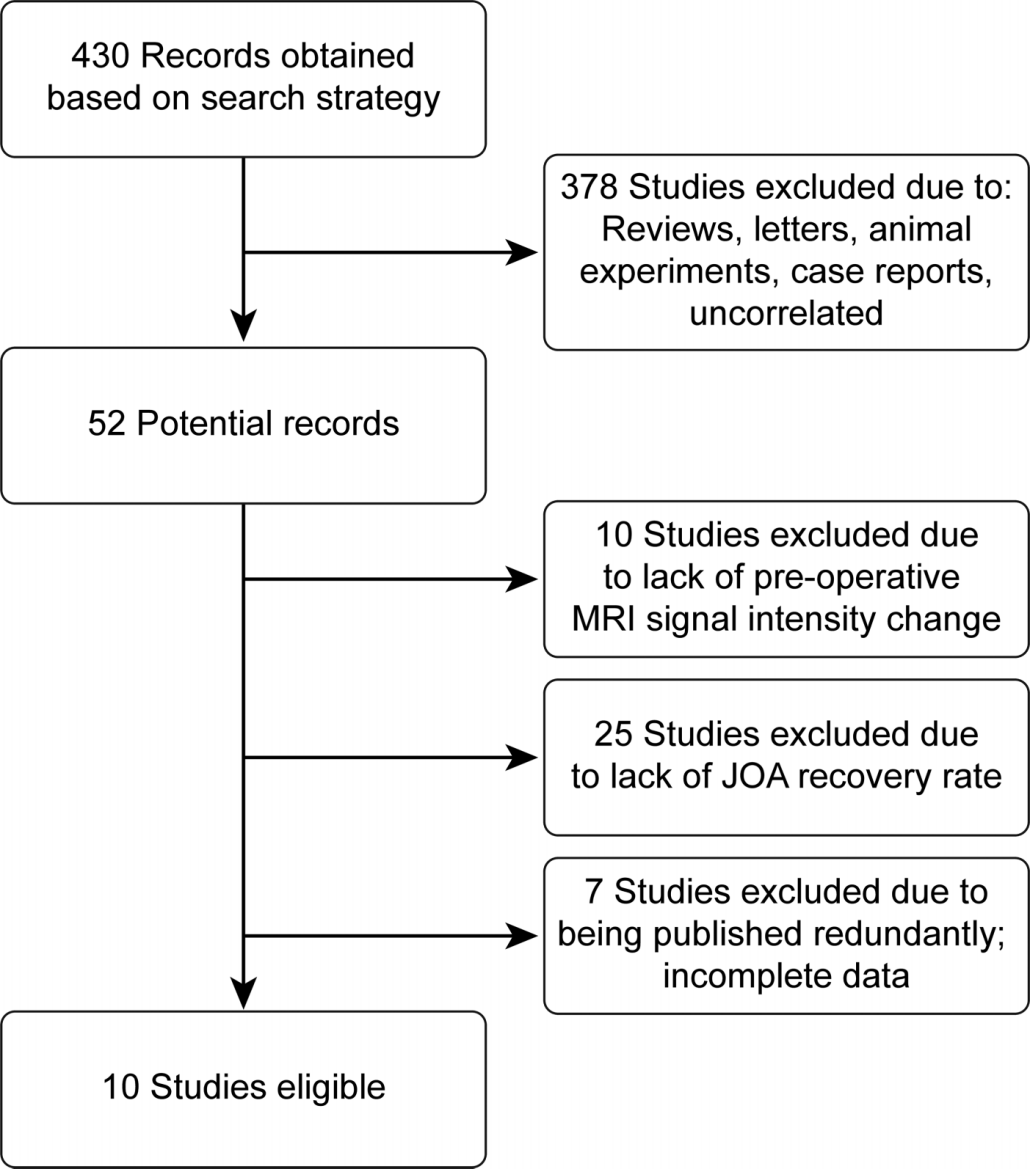
The search strategy.

The search strategy (Figure 1) identified 430 potential studies from the Medline database and the Cochrane Central Register of Controlled Trials. In total, 413 papers were excluded according to our inclusion criteria. Among those excluded papers, 8 studies used the modified JOA (mJOA) RR, the Neck Disability Index (NDI), and the Nurick grade to estimate the surgical outcome; for these studies, we performed a descriptive analysis. Seven additional studies were excluded from the analysis because of redundant publication or incomplete data. Therefore, 10 articles were ultimately included 5-6,.

### Assessment of risk of bias

All of the eligible articles were non-randomized and the MINORS score was used to assess the risk of bias. The scores varied from 16 to 21 (Table 1). Considering the previously published papers scoring >75% of the maximum score 18, designated as high quality, there were eight high-quality articles.

### Study characteristics

Basic information on the included articles is presented in Table 1. Of the 10 studies, 4 did not provide any data about disease course 10,11,13,15, and 1 did not mention the mean follow-up duration 11. The postoperative JOA RR was used to estimate the surgical outcome in all selected studies.

### T2-weighted MRI ISCs (+) *versus* ISCs (-)

**Figure 2-:**
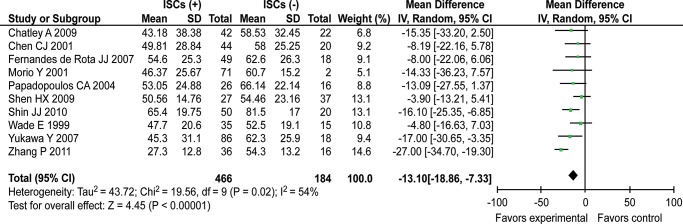
The forest plot of T2-weighted magnetic resonance imaging intramedullary signal changes (+) *versus* intramedullary signal changes (-).

In the 10 studies, 466 patients were included in the T2-weighted image ISCs (+) group and 184 were included in the T2-weighted image ISCs (-) group. Overall, the WMD was statistically significant (WMD=-13.10, *p*<0.00001, 95% CI: -18.86 to -7.33) in favor of the ISCs (-) group. The forest plot showed there was statistically significant heterogeneity (*I^2^*=54%, *p*=0.02) (Figure 2).

### Subgroup analysis

**Figure 3-:**
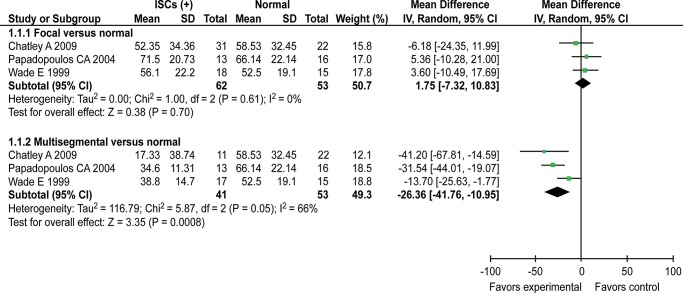
The forest plot of the focal, multisegmental group *versus* the normal group.

Three studies divided the T2 hyperintensity changes into focal and multisegmental changes 5,11,12. The WMD was equivalent for the focal and normal groups (WMD=1.75, *p*=0.70, 95% CI: -7.32 to 10.83). Statistical heterogeneity was not detected among the studies (*I^2^*=0%, *p*=0.61). Compared with the normal group, pooled estimates showed that the multisegmental group achieved a significantly poorer surgical outcome (WMD=-26.36, *p*=0.0008, 95% CI: -41.76 to -10.95). Statistically significant heterogeneity was clearly present (*I^2^*=66%, *p=*0.05) (Figure 3).

**Figure 4-:**
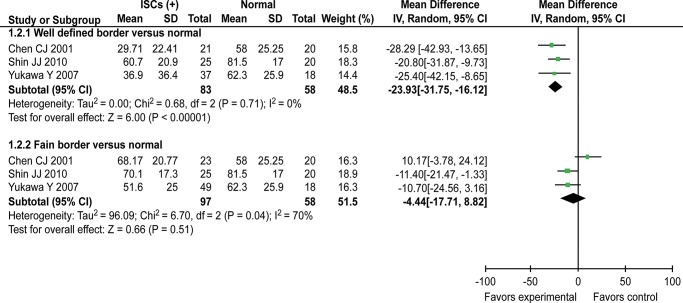
The forest plot of the faint, well-defined border group *versus* the normal group.

Relevant data about faint and well-defined border signal intensity changes on T2-weighted images were documented in three articles 9,13,16. Overall, there was no significant difference between the faint border and the normal groups (WMD=-4.44, *p*=0.51, 95% CI:-17.71 to 8.82). Significant heterogeneity was detected (*I^2^*=70%, *p*=0.04). Compared with the normal group, pooled data indicated a poorer surgical outcome in the well-defined border group (WMD=-23.93, *p*<0.00001, 95% CI: -31.75 to -16.12). There was no evidence of statistically significant heterogeneity *(I^2^*=0%, *p*=0.71) (Figure 4).

Of the included studies, only 1 study reported postoperative JOA RRs for patients with low signal intensity changes on T1-weighted images 15. The data revealed that this group had a poorer prognosis (WMD=-41.60, *p*=0.008, 95% CI: -72.31 to -10.89).

### Descriptive analysis

Eight studies used the mJOA RR, the NDI and the Nurick grade to estimate the surgical outcome; for these studies, we performed a descriptive analysis 18,. Six records studied both T1 and T2 ISCs and 2 records only studied T2 ISCs. The conclusions were that both T2 ISCs and especially the signal changes that were multisegmental and intense and T1 ISCs indicated a poor prognosis (Table 2).

## DISCUSSION

According to previous studies, signal intensity changes in the spinal cord on MRI images could reflect pathologic changes. Due to advances in MRI techniques and software, we can now detect various degrees of signal intensity changes in CSM patients. Recently, a correlation between MRI findings and clinical recovery after therapy has been widely discussed 4,5,6,7,8,9,10,11,12,13,[Bibr b16-cln_71p179]16^17^,^18^,^19^,^20^,^21^,^22^,^23^,^24^25^26^. However, at the same time, no significant difference in MRI parameters has been noted. We believed that comparison between previous articles would be meaningful.

Since Takahashi et al. 1 first described high signal intensity on T2-weighted MRI of the spinal cord in patients with CSM, many studies have investigated the association between high-intensity signal changes on T2-weighted images and surgical outcomes. However, the prognostic value of ISCs on T2-weighted MRI remains controversial: several authors 5,13,15,17 observed that ISCs were not related to poor outcomes in patients with CSM, whereas others 18,19 reported that patients with ISCs had a poor prognosis after surgery. Our meta-analysis revealed that patients with ISCs on T2-weighted images had a poorer outcome compared with patients with no signal intensity changes.

In certain studies, the signal changes were further subdivided. Wada et al. 5 divided patients with ISCs on T2-weighted MRI into two groups according to the level involved: one group had ISCs located at one level and one group had ISCs that extended into two or more levels. The authors found that patients with multisegmental ISCs tended to have poorer outcomes and that those with focal ISCs had a similar outcome as those without ISCs. Chen et al. 13 proposed another classification, subdividing ISCs on T2-weighted MRI into two patterns: ISCs with a faint, light border and ISCs with an intense, well-defined border. Light ISCs reflected mild neuropathologic alteration in the spinal cord and greater recuperative potential, whereas intense ISCs reflected severe alteration and less recuperative potential. It seemed that only intense ISCs corresponded to a poor prognosis. Our meta-analysis revealed that multisegmental and intense ISCs indicated worse surgical outcomes than focal and light ISCs did.

Many studies have investigated the significance of low signal intensity on T1-weighted MRI, which is considered to reflect pathologically irreversible changes 14,15,18. Fernandes de Rota et al. 14 reported that low signal intensity changes presented only in patients who had already had high T2 signal intensity changes and most of the changes were multisegmental. Morio et al. 15 found that low signal intensity changes on T1-weighted sequences indicated a poor prognosis and that high signal intensity changes on T2-weighted sequences reflected a broad spectrum of spinal cord recuperative potential.

Other factors, such as the age at the time of the operation, symptom duration, narrowing of the spinal canal and preoperative cervical lordosis, have been reported to influence the surgical outcome in patients with CSM 20,21,22,23,24. Moreover, Arvin et al. 25 revealed that findings on postoperative MRI were of predictive value in determining outcomes in CSM patients.

There are several limitations in the present meta-analysis. First, the best evidence consists of a meta-analysis of numerous randomized controlled trials with high quality, but 10 articles included in this study were non-randomized. Second, heterogeneity was detected among the studies when we pooled the outcomes. This heterogeneity could be explained by the various study qualities, study designs and patient baseline data. Third, we only discussed the significance of preoperative MRI imaging in predicting postoperative recovery, but many other factors related to surgical outcomes were not strictly controlled. Thus, further larger-sample trials with high study quality will still be essential to clarify the prognostic value of MRI signal changes in patients with CMS.

In summary, the results of this meta-analysis showed that the postoperative JOA RR was poor in patients with both intramedullary signal intensity changes on T2-weighted MRI of the spinal cord, especially when the ISCs were multisegmental, had a well-defined border and were intense, and low signal intensity on T1-weighted MRI. In summary, preoperative MRI imaging can be used to predict postoperative recovery in patients with CSM.

## COMMENTS

As the cause and the pathogenesis of signal intensity changes within the spinal cord remain unclear, the significance of the signal intensity changes for prognosis remains controversial. This meta-analysis was thus performed to elucidate whether preoperative signal intensity changes could predict surgical outcomes in patients with cervical spondylosis myelopathy on the basis of T2-weighted and T1-weighted magnetic resonance imaging images.

## AUTHOR CONTRIBUTIONS

Chen H was responsible for the study design and the manuscript preparation. Nisar M, Zeng HB and Dai LF collected and analyzed the data. Chen H and Pan J were the principal investigators of the study.

Xiang GH was responsible for the study design and the manuscript finalization. All authors read and approved the final manuscript.

## ACKNOWLEDGMENTS

We thank Long Chen and Haidong Jin for their comments and advice.

## Figures and Tables

**Table 1:** Characteristics of the included studies.

Study	Year	Quality scale*	Cases (n)	Mean age (years)	Disease course (months)	Gender (M:W)	Mean follow-up (months)
Chatley et al.	2009	19/24	64	47.1	10.26	57:7	>6
Chen et al.	2001	21/24	64	56.67	NA	42:22	>6
Fernandes de Rota et al.	2007	20/24	67	59.5	24.1	50:17	39
Morio et al.	2001	21/24	73	64.0	NA	50:23	40.8
Papadopoulos et al.	2004	16/24	42	57.5	NA	27:15	NA
Shen et al.	2009	18/24	64	58.5	NA	46:18	34
Shin et al.	2010	18/24	70	51.1	2.5	45:25	32.7
Wade et al.	1999	17/24	50	61.0	9.1	36:14	35.1
Yukawa et al.	2007	21/24	104	61.0	20	67:37	>12
Zhang et al.	2011	19/24	52	56.3	16.1	30:22	23

**<?ENTCHAR ast?>:** The quality of the included studies was assessed using the MINORS score.

*NA*, not available.

**Table 2:** Descriptive analysis.

Study	Cases (n)	Mean age (years)	Mean follow-up (years)	Duration of symptoms (months)	Outcome variable(s)	MRI signal intensity studied	Conclusions
Arvin et al.	57	55.54	12.0	30.27	mJOA RR, NDI, Nurick grade	T1 and T2 ISCs	The presence of a low T1 signal, a focal increased T2 signal and segmentation of T2 signal changes indicated a poorer outcome.
Yagi et al.	71	62.9	60.6	13.2	JOA RR	T1 and T2 ISCs	The presence of ISCs on T1 as well as T2 and postoperative expansion of ISCs indicated a poor long-term prognosis.
Mastronardi et al.	47	54.0	40.2	11.5	Nurick grade, mJOA score	T1 and T2 ISCs	T1 and T2 ISCs indicated the worst prognosis, whereas the regression of T2 ISCs was associated with a better prognosis.
Suri et al.	146	47.1	NA	11.7	Nurick grade	T1 and T2 ISCs	The patients without ISCs or with ISCs on only T2 had a better outcome than the patients with ISCs on both T1 and T2.
Avadhani et al	35	57.8	51.3	9.3	Nurick grade RR	T1 and T2 ISCs	ISCs on both T1-weighted imaging and T2-weighted imaging were more predictive of the surgical outcome than ISCs only on T2.
Alafifi et al.	76	61.8	30.0	6.5	Nurick grade	T1 and T2 ISCs	Low ISCs on T1 indicated a poor prognosis, whereas ISCs on T2 did not.
Vedantam et al.	197	48.8	35.2	8.0	Nurick grade	T2 ISCs	Intense ISCs were associated with a lower probability of cure.
Park et al.	80	62.1	NA	19.1	NCSS	T2 ISCs	Multisegmental ISCs were independently associated with a poorer NCSS recovery rate.

mJOA score: modified Japanese Orthopaedic Association score, NDI: Neck Disability Index, NCSS: Neurosurgical Cervical Spine Score, Nurick grade RR = (postoperative modified Nurick Score-preoperative modified Nurick Score)/(6-preoperative modified Nurick Score) × 100.
